# Linking communities and health facilities to improve child health in low-resource settings: a systematic review

**DOI:** 10.1093/heapol/czae028

**Published:** 2024-04-15

**Authors:** Agnese Iuliano, Rochelle Ann Burgess, Funmilayo Shittu, Carina King, Ayobami Adebayo Bakare, Paula Valentine, Ibrahim Haruna, Tim Colbourn

**Affiliations:** Institute for Global Health, University College London, 30 Guilford Street, London WC1N 1EH, United Kingdom; Institute for Global Health, University College London, 30 Guilford Street, London WC1N 1EH, United Kingdom; Department of Paediatrics, University of Ibadan, CW23+FJV University College Hospital, Queen Elizabeth I I Road, Agodi, Ibadan, Oyo 00285, Nigeria; Department of Global Public Health, Karolinska Institutet, Norrbackagatan 4, Stockholm 171 76, Sweden; Institute for Global Health, University College London, 30 Guilford Street, London WC1N 1EH, United Kingdom; Department of Global Public Health, Karolinska Institutet, Norrbackagatan 4, Stockholm 171 76, Sweden; Department of Global Public Health, Karolinska Institutet, Norrbackagatan 4, Stockholm 171 76, Sweden; Department of Community Medicine, University of Ibadan, CW22+H4W, Queen Elizabeth I I Road, Agodi, Ibadan, Oyo 200285, Nigeria; Save the Children, 1 St John’s Ln, London EC1M 4AR, United Kingdom; Save the Children International, Plot 773 Cadastral Zone B03, Wuye District, Ankuru 902101, Nigeria; Institute for Global Health, University College London, 30 Guilford Street, London WC1N 1EH, United Kingdom

**Keywords:** Community-facility link, quality of care, under-5 children, community empowerment, development committees, community health workers, realist principles

## Abstract

Community–facility linkage interventions are gaining popularity as a way to improve community health in low-income settings. Their aim is to create/strengthen a relationship between community members and local healthcare providers. Representatives from both groups can address health issues together, overcome trust problems, potentially leading to participants’ empowerment to be responsible for their own health. This can be achieved via different approaches. We conducted a systematic literature review to explore how this type of intervention has been implemented in rural and low or lower-middle-income countries, its various features and how/if it has helped to improve child health in these settings. Publications from three electronic databases (Web of Science, PubMed and Embase) up to 03 February 2022 were screened, with 14 papers meeting the inclusion criteria (rural setting in low/lower-middle-income countries, presence of a community–facility linkage component, outcomes of interest related to under-5 children’s health, peer-reviewed articles containing original data written in English). We used Rosato’s integrated conceptual framework for community participation to assess the transformative and community-empowering capacities of the interventions, and realist principles to synthesize the outcomes. The results of this analysis highlight which conditions can lead to the success of this type of intervention: active inclusion of hard-to-reach groups, involvement of community members in implementation’s decisions, activities tailored to the actual needs of interventions’ contexts and usage of mixed methods for a comprehensive evaluation. These lessons informed the design of a community–facility linkage intervention and offer a framework to inform the development of monitoring and evaluation plans for future implementations.

Key Messages

- Community–facility linkage interventions can improve community health in low-income settings by creating/strengthening a relationship between community members and local healthcare providers. However, there is no definitive evidence in the literature on the effectiveness of community–facility linkage interventions, and we aimed to fill this gap.
- We tried to understand which features are associated with better child health outcomes and which characteristics are linked with improved community participation when implemented in rural areas of low/lower-middle-income countries. Key lessons for success are: (1) the active inclusion of hard-to-reach groups, (2) the involvement of community members in programmatic decisions, (3) the focus on the actual context of the intervention and (4) the use of mixed methods for evaluation.
- We built an evaluation framework that can be used to assess the impact of community-based and facility link interventions.
- We provided an angle to analyse community–facility linkage interventions, trying to shed light on key features for success. We hope this will influence potential new implementations and that our framework will serve as a guide for future evaluation plans.

## INTRODUCTION

With 5 million under-5 deaths in 2021, childhood mortality still presents a major health challenge globally (UNICEF, 2023). Local, national and international bodies, including local government and NGOs, have implemented a wide variety of interventions focused on health systems’ improvement to try to reduce these avoidable deaths. This is especially true in resource-constrained settings where child mortality is higher (Cha and Jin, 2020; World Health Organization, 2023). Causes of this burden include poor healthcare provision at local health centres (Wanjau *et al*., 2012; Kruk *et al*., 2018), and poor health knowledge and trust towards the healthcare system at community levels (Woskie and Fallah, 2019; Moucheraud *et al*., 2021). Therefore, these improvement interventions have focused on all system levels, from facility improvements to community health education. Often, development programmes work at the community and facility level at the same time, trying to tackle health challenges from multiple perspectives (Metwally *et al*., 2020).

Frequently, these types of programmes do not focus on creating a connection between the two different locations of action, i.e. facility-based and community-based interventions remain confined to their settings. This therefore hinders the possibility of obtaining sustainable and long-term improvements (Oguntunde *et al*., 2018). However, whenever community and facility interventions are implemented, a community–facility linkage component could be included in the delivery plan. Community–facility linkage is defined as a ‘formalized connection between a health facility and the communities it serves to support improved health outcomes’ (Gulaid, 2015, p.7). There is no definitive evidence in the literature regarding the effectiveness of interventions aimed at strengthening such connections, but numerous approaches could foster accountability improvement for health in local communities, reduce missed opportunities for adequate care, and improve appropriate and timely care-seeking behaviours and overall health outcomes (World Health Organization, 2008; Diaconu *et al*., 2020).

One feature that this type of ‘whole system’ implementation could benefit from is the more active participation of local communities in the intervention design and delivery. Their knowledge of the health challenges peculiar to the intervention setting could help to tailor interventions to the population’s actual needs (World Health Organization, 2016). Also, empowering communities to take ownership and lead development programmes is considered a powerful tool to obtain a long-lasting transformation, sustainable over time, maintaining positive outcomes after the intervention is concluded (Bigdeli *et al*., 2020).

As these interventions can vary a lot in how they are implemented and the evidence of effect is mixed, we conducted a systematic literature review to identify the components that make them successful, or not. Our research focused on answering this research question: ‘what key features (contexts, mechanisms, strengths, weaknesses, limitations) shape outcomes of previous community-facility linkage interventions to improve child health in rural areas of low or lower-middle income countries (similar to the socio-economic and geographical conditions in Jigawa, Nigeria)?’. Our aim was to understand which attributes contribute to better child health outcomes and to illuminate which characteristics are linked with improved community participation when implemented in specific settings. While a prior literature review explored community participation and health facility committees as a mean to connect communities with health facilities (McCoy *et al*., 2012), there is a gap in the existing research. To the best of our knowledge, there has been no comprehensive review specifically addressing various types of community–facility linkage interventions and their impact on child health. This review was conducted with the purpose of informing the theory and design of a whole systems intervention being trialled in Jigawa State, Nigeria, with the specific aim of supporting the design of the monitoring and evaluation plan for the community–facility linkage component of it [INSPIRING Project: (ISRCTN39213655, 2019; King *et al*., 2021)]. This intervention aims to reduce childhood mortality due to pneumonia and other infectious diseases in a setting where the under-5 mortality rate is 192 deaths per 1000 live births (National Population Commission—NPC and ICF, 2019), and there is low-quality healthcare system, poor community health education and lack of protective and preventive factors (Iuliano *et al*., 2020; King *et al*., 2020; Shittu *et al*., 2020).

## Methodology

### Search strategy

We conducted a systematic literature review examining three online databases (Web of Science, PubMed and Embase). By utilizing these databases, we aimed to ensure a thorough and multidisciplinary approach to our research topic, allowing us to gather a comprehensive overview of the existing literature, capturing a wide range of relevant studies and ensuring the validity and reliability of our findings. We used these search terms: health facility/hospital, community-based organizations/community health workers/community development organizations, linkage/referral, child/paediatrics (specific search strategy for each database is detailed in Appendix 1). We did not apply any additional filter in our search at this stage. The search strategy was peer-reviewed by the INSPIRING research team with the technical support of the librarian team of the authors’ institute. After searching the online databases, we obtained records of the articles to screen for studies published by 3 February 2022.

### Inclusion/exclusion criteria

The variables included in the selection criteria were chosen to find interventions with similarities to the ones being implemented in the INSPIRING Jigawa trial (King *et al*., 2021). As Jigawa is predominantly non-urban, and Nigeria is a lower-middle-income country (WorldBank, 2021), two of the inclusion criteria imposed that the studies had to be set in rural areas (thereby excluding those set in urban environments) and in low- or lower-middle income (LMIC) countries. The intervention being evaluated had to have a community–facility linkage component—which was defined as: any form of intermediation or connection between the health facility and the community it served. This linkage could be established through mechanisms such as community health workers, health development committees, or other formalized bodies or activities aimed at bridging the gap between communities and health centres, specifically with the goal of enhancing under-5 child health. We specifically sought interventions that went beyond mere focus on health facility strengthening or community health promotion; studies emphasizing these aspects exclusively were excluded. While these activities could coexist, a crucial criterion was the presence of a connecting factor that facilitated collaboration between health facilities and community initiatives.

Apart from an interest in the characteristics of the interventions assessed in the articles, the outcomes of interest had to be related to under-5 children’s health, and included: under-5 mortality, care-seeking behaviour, healthcare attendance rates and relationship between communities and healthcare providers.

Other inclusion criteria were: peer-reviewed articles (to ensure high quality, reliability and rigour in our systematic review) containing original data and written in English (due to limitation in translation capabilities). We did not apply any date restriction or restriction on study design used for intervention evaluation.

### Selection of studies, data extraction and risk of bias assessment

Following PRISMA reporting guidelines (Page *et al*., 2021), the first step involved conducting an analysis of all the found articles’ titles to discover those to potentially include. In the second step, all the abstracts of the articles selected based on the title were scrutinized. Third, a full-text analysis of all the abstract-based potentially relevant studies was performed. The final choice of the articles to include was made based on whether the intervention methodology and outcome data were deemed fitting the predefined inclusion/exclusion criteria, and whether the papers met quality standards (any limitations were noted and are reported in the Results table). The first and last author independently conducted the same screening process. Once they each reached the final stage of selecting papers for inclusion, the two authors met and resolved in discussion any disagreement on eligibility. We did not register the review, and we decided not to use any bias assessment tools. The variety in methodologies of the included articles would have required the usage of multiple checklists, and we deemed that a critique of each paper’s quality by the authors, who have methodological expertise to assess the articles, suffices, along with the fact that the included papers have already been peer reviewed. We carefully evaluated each study’s methodology and potential of bias against the limitations reported by the papers and our own assessment.

### Analysis

In order to analyse previous interventions based not only on their characteristics and successes, but also on their transformative and community-empowering capacities, we used Rosato’s integrated conceptual framework for community interventions (Rosato, 2015). Any intervention can be assessed based on nine practice variables. Each of these variables is classified using roman numerals from I (1) to V (5), the more community-driven and centred, the higher the ranking (with the following variable descriptions not being mutually exclusive, but rather each new classification adding to the previous ones):

- *Conceptualization of health* [how the intervention defines health, from the medical model (‘absence of diseases and disorders’) to the behavioural model (‘product of healthy lifestyle choices’), to the socio-environmental model (‘product of social, economic, and environmental determinants’)].
- *Goal* (what the intervention goal in relation to health is: from simple eradication of health problems affecting individuals to changes in health knowledge and behaviour in groups in the community to increases in community capacities to change the political system).
- *Target group* (who the intervention is targeting for improvements in health: from the entire community to the same community but within that some specific marginalized subparts, to functional subparts of the community, defined by shared needs, that are the most marginalized and suffer at the hands of the wider community).
- *Existing strengths and weaknesses* and
- *Role of external agent* (how much the intervention recognizes and builds on the existing capabilities of the community to address health issues and what the role of the external agent in the intervention is: from a community considered weak and lacking capacities to help itself, therefore needing the external agent to take all responsibilities, to a community lacking some skills but in possession of some other capacities—an external agent needed to marshal the existing ones—to a community considered to be capable of solving its health problems, with the external agent only reinforcing these capacities).
- *Participation* (involvement of the community in decision-making about the intervention: from being simply informed to being consulted tokenistically, to joint decision-making with external agents, to the community leading the decision-making process and being accountable for the outcomes).
- *Role of community* (from being the passive setting/target of the intervention, to being the active source of solutions for the intervention, to being the active agent of change, defining its own health problems, setting its own agenda and being the source of solutions).
- *Tools and methods* (what the intervention employs, from clinical health methods in a short timeframe to behaviour-change ones, to social capacity-building methods in a longer period).
- *Resources* (responsibility of mobilizing resources from laying on the external agent, to being equally split between the external agent and the community, to being entirely in the hands of the community, with the external agent only supporting them and brokering access to the resources).

Five statements representing different levels on the intervention continuum were created for each of the nine practice variables by Rosato. We identified the most fitting statement that reflected each paper’s practice concerning each variable and assigned scores. We then plotted these scores on spider diagrams, facilitating a visual representation of the results.

We also applied realist principles to synthesize the included papers. According to realist methodology (Pawson *et al*., 2005; Pawson, 2013), there is a causal path to explain how an intervention works, what makes it successful or why it fails. Starting from a programme theory to justify the intervention plan, then Context (the physical and social environment) and Mechanisms [*resources* offered by the programme and how they are received and resonate among the participants—*reasoning* (Dalkin *et al*., 2015)] lead to specific Outcomes (CMO configuration). Through this iterative process, a refined understanding of the intervention’s underlying mechanisms and contextual factors emerges. This leads to the development of a Middle Range Theory (MRT) (Pawson, 2017), which serves as a specific and contextually grounded framework. While the original programme theory remains the foundation, the insights gained through the realist analysis process enhance the theoretical framework and the MRT becomes a new starting point for future implementations.

We aimed to build a monitoring and evaluation tool to assess the community–facility linkage component of future projects. So, we gathered the points in common that the MRTs of the interventions included in this review showed and used them to build a new framework to inform future analysis plans.

Furthermore, our study has an interest in foregrounding transformative principles in our research and implementation practices. Mertens (1999, 2007) has argued research and evaluation praxis is more effective when decision making, planning and implementation include direct involvement of communities—instead of leaving them as mere targets of interventions—making change more plausible and sustainable. As such, our assessment of the papers included in this review also sought to interrogate the extent to which the existing literature speaks to these principles.

## Results

**Figure 1.**
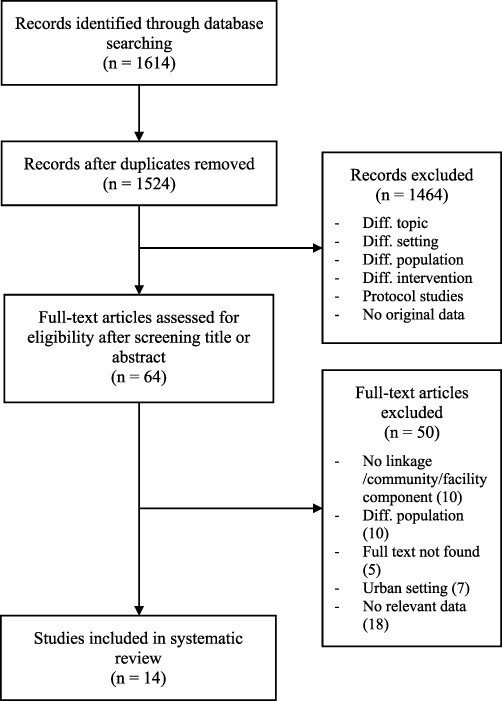
Flow diagram of the literature review process

A total of 1528 publications were identified through the online databases, after duplicate elimination. Following title and abstract screening, we extracted 64 studies for full-text review. Fourteen papers met the inclusion criteria and presented relevant data (Bari *et al*., 2006; Björkman and Svensson, 2009; Olayo *et al*., 2014; Waiswa *et al*., 2015; Cannon *et al*., 2017; Gupta *et al*., 2017; Pol *et al*., 2017; Kamugisha *et al*., 2018; Oguntunde *et al*., 2018; Tancred *et al*., 2018; Awasthi *et al*., 2019; Kushitor *et al*., 2019; Ndaba *et al*., 2020; Var *et al*., 2020) (Figure 1).

### Study descriptions

Characteristics of included studies are presented in Table 1. Of the 14 studies, 9 were set in Sub-Saharan Africa [Nigeria (Cannon *et al*., 2017; Oguntunde *et al*., 2018), Tanzania (Tancred *et al*., 2018), Uganda (Björkman and Svensson, 2009; Waiswa *et al*., 2015; Kamugisha *et al*., 2018; Tancred *et al*., 2018), Ghana (Kushitor *et al*., 2019), Kenya (Olayo *et al*., 2014), South Africa (Ndaba *et al*., 2020)], and 5 in south Asia (India (Gupta *et al*., 2017; Awasthi *et al*., 2019) Cambodia (Pol *et al*., 2017; Var *et al*., 2020) and Bangladesh (Bari *et al*., 2006).

**Table 1.** Studies characteristics

Reference	National site	Sub-national site	Study design	Study population	Quant. analysis	Qual. analysis	Data source	Int. characteristics	Int. duration	Data coll. time	Outcomes of interest	Limitations
Oguntunde *et al*., 2018	Nigeria	Jigawa, Kaduna, Kano (NW)	Mixed method	- Facility DC members - Facility HP - Facility clients	Y	Y	- Surveys and FGDs with FDC members - In-depth interviews HPs - Exit interviews with facility clients	Facility DCs [one facility HP and 15 community residents (from all ethnic, religious, age and gender groups)]	2010–2015	2016	Perceptions regarding FDCs: - role and responsibilities - utility in improving QoC and FSs - how they could be impr.	- No control data - No before/after data
Cannon *et al*., 2017	Nigeria	Sokoto state	Case study	- Mothers (used drug) - Drug keepers - CHVs - HPs - Husbands - WDC members	N	Y	- Key informant interviews - FGD	c-b int. to reduce maternal and newborn deaths: - Misoprostol to women after childbirth to prevent haemorrhage - Chlorhexidine to newborns to reduce cord infection Distribution chain system involving LGA government, WDC, CHV	2009–2015	2013	- Perceived successes and benefits of using these drugs by key stakeholders - Key factors for the success - Barriers - Key lessons learned	- Selection bias (convenience sampling approach) - Self-reported data (may be biased)
Awasthi *et al*., 2019	India	Lucknow Uttar Pradesh	Before and after	Mothers of children 2–59 months old	Y	N	- Baseline survey - Endline survey	Pneumonia Awareness Sessions (PAS): - educational messages/sessions for caregivers at village (by CHVs) and facility (by nurse/midwife) level - infrastructure strengthening (equipment, staff, drugs) - HP training	2015–2018	2016–2018	Changes in: - caregivers’ pneumonia knowledge - csb	- Small number of clusters - Proximity of int. study blocks
Tancred *et al*., 2018	Tanzania and Uganda	Tandahimba (Tz)Mayuge (Ug)	Qualitative process evaluation	- Caregivers - CHVs - CHVs mentors - Village leaders - HF staff	N	Y	- In-depth interviews - Birth narratives - FGDs	‘Expanded Quality Management Using Information Power to improve Maternal and Newborn Health’ (EQUIP) int.: - Quality Improvement (QI) processes at community, facility, and district levels via CHVs through plan-do-study-act cycle (PDSA)	2011–2014	2012–2013	Changes in: - demand for maternal and newborn health FSs - community-level maternal and newborn care practices	- Tanzania: data coll. only in four villages - Uganda: data less comprehensive - Data collected before the int. ended
Kushitor *et al*., 2019	Ghana	Gushiegu, Kumbungu; Central Tongu, Nkwanta South	Qual. evaluation for prospect scale-up	- Caregivers of U5 children - Male and female adolescents - Community leaders	N	Y	FGD with CMs	Community Health Planning and Services (CHPS) initiative: - PHC local provision by Community Health Officers in health posts			- Community perceptions of CHPS - Community involvement - Ways to impr. CHPS	- High ranking for CHPS might be biased due to fear of FSs withdrawal
Var *et al*., 2020	Cambodia	Takeo province	Cluster-randomized stepped wedge trial	- Mothers who gave birth in included HFs - HPs (chief, midwives, other professional staff) - Village Health Support Group (VHSG) from included HFs	Y	N	Questionnaires: - last trimester of pregnancy - 14 days after birth - 28 days after birth	Newborn Infection Control and Care Initiative (NICCI): - HFs: staff training, hygiene practices, referral, data recording - community: VHSG + CHVs to impr. hygiene, danger signs recognition, referral - household: impr. hygiene, breastfeeding, danger signs recognition, referral	2015–2016	2015–2016	- Recognition of newborn danger signs by caregivers and VHSG - Changes in csb - Time reduction between symptoms onset and referral - QoC from HPs	- Various variables self-reported (hygiene, h.care staff) - Selection bias (women who did not deliver in the selected facilities excluded) - Small sample size (impossible to evaluate mortality rate changes)
Olayo *et al*., 2014	Kenya	Butere district, Kisumu district, Garissa district	Quasi-experimental	Mothers and caregivers	Y	N	Pre-/post-int. surveys in int. and control sites	Community Health Strategy (CHS): - formation of DCs at community and HF levels - formation and training of CHWs - dialogues on progress and changes needed with community/HPs (dialogue > plan > act > assess > dialogue)	2011–2012	2010–2012	- Immunization coverage - Ante-natal visits - HF delivery - Insecticide-treated nets use - Water and sanitation - Food availability - Clinic cards	- Selection of int. and control sites (other enabling factors in int. sites)
Kamugisha *et al*., 2018	Uganda	Kanungu district	Retrospective cross-sectional study	Children born between Jan. 2015 and Dec. 2016	Y	N	Nurses and CHVs’ book records of household visits and child outcome	Health for All (HEAL): - nurses: antenatal/postnatal visits plus health education - CHVs: households visit to do health promotion, education and referral to HF (with daily discussions on how to address health issues)	2014–2016	2014–2016	Changes in: - ANC attendance - supervised deliveries - completion of immunization cycles - earlier infant illness recognition/treatment- malnutrition - number of infant deaths	- No formal data collection (no evaluation plan) - No control area- No activities analysis - Lack of work also on education and financial points
Pol *et al*., 2017	Cambodia	Siem Reap	Mixed methods	- CMs (caregivers, children) - healthcare workers	Y	Y	- Questionnaires - Event logs - Reflective interviews	QoC impr. via: - Science café’ (SC): bi-monthly meetings with HPs and caregivers in cafes to discuss/share suggestions on health topics informally - Young Persons Advisory Group (YPAG): engage children aged 10–15 years, get their opinion on hospital care delivery, raise awareness	2015-ongoing at publication time	2017	Changes in: - caregivers’ knowledge of child health - participation in hospital activities - relationship between community and facility members	- Responses are only from study participants (might not reflect the entire community) - Participants are self-selected - Evaluation is early-stage
Gupta *et al*., 2017	India	Haryana	Mixed methods	- CMs (mothers, caregivers) - HPs (medics, nurses, midwives) - Social health activists - Community leaders	Y	Y	- District Level Household Surveys (DLHS) - FGD - In-depth interviews	- Infrastructure impr. (free drugs, transport, medical mobile units) - Cash benefits for women (free delivery and free sick neonate’s treatment) - Establishment of social health activists (SHA) (local women to do health promotion, education)	2005/06-2012/13	2013	- Health system strengthening - Communitization (community education and empowerment) - Reducing geographical and socio-economic disparities in maternal and child health	- No control region - Confounding factors (there might be other development programmes in place)
Bari *et al*., 2006	Bangladesh	Mirzapur upazila	Cross-sectional study	- Pregnant women- Newborn children	Y	N	- Baseline/ primary-secondary adequacy questionnaires - CHW’s records - Referral hospital registers	Package of maternal and newborn care: - CHW training and home visits before/after delivery (basic treatments provided or referral to the HF) - families health education - neonatal care strengthening at HF	2003–2005	2003–2005	Csb changes	Unclear why self-referral increased so much
Waiswa *et al*., 2015	Uganda	Iganga and Mayuge districts	Cluster-randomized control trial	- Pregnant women - Newborn children	Y	N	- Baseline/endline survey	- CHW home visit package (pregnancy and newborn care) - HF strengthening (equipment provision, staff training)	2009–2011	2008–2011	- Coverage of ANC FSs impr. - Skilled attendance at delivery - Postnatal care - Breastfeeding, thermal care, and hygiene practices impr.	- Prone to recall and selection bias - CHWs not equipped to monitor weight of newborns (LBW great risk factor for mortality) - Study not powered to detect mortality differences
Ndaba *et al*., 2020	South Africa	KwaZulu-Natal province	Qualitative exploratory research	- Comm. leaders - Comm. members - Healthcare providers	N	Y	- Notes from training sessions and meetings - Observation of CAG activities - Meetings’ minutes	Establishment of Comm. Advisory Groups (CAGs): public forum to discuss comm. issues/concerns with healthcare providers and build a Comm.-facility partnership to improve quality of care			CAG: - establishment - activities - accomplishments	- No formal systematic data analysis (rapid assessment) - Implementation in only one administrative ward - Challenges to reach isolated communities
Björkman and Svensson, 2009	Uganda	50 facilities in rural areas from 9 districts	Randomized control trial	- HP - Comm. members	Y	N	- HP survey - Household survey	Citizen reports cards: enhance comm. involvement and monitoring of the delivery of PHC using Participatory Rural Appraisal (PRA) methodology (analysis of comm.’s environment and identification, discussion and solution-finding of problems).	2005–2006	2004–2006	- Changes in quantity and quality of health care - Improved child health outcomes - Behaviour change of HP - Comm. engagement	- Preliminary results, so unsure about sustainability of the project - Data collection tools limited—to evaluate how to expand them

Abbreviations: Y = Y N= no; coll. = collection; c-b = Community-based; comm. = community; CHV = Community Health Volunteer; CHW = Community Health Worker; csb = care-seeking behaviour; DC = Development Committee; FGD = Focus Group Discussion; FS = Facility Services; HF = Health Facility; HP = Healthcare Providers; impr. = improvement; int. = intervention; QoC = Quality of Care; qual. = qualitative; quant. = quantitative.

Overall, three studies had a mixed-method design (Gupta *et al*., 2017; Pol *et al*., 2017; Oguntunde *et al*., 2018), four were solely qualitative [a case study (Cannon *et al*., 2017) and three process evaluations (Tancred *et al*., 2018; Kushitor *et al*., 2019; Ndaba *et al*., 2020)], while the other seven publications presented quantitative data exclusively (Bari *et al*., 2006; Björkman and Svensson, 2009; Olayo *et al*., 2014; Waiswa *et al*., 2015; Kamugisha *et al*., 2018; Awasthi *et al*., 2019; Var *et al*., 2020).

Studies included a wide range of interventions to link communities and facilities, groupable into two main categories: development committees and community health workers/volunteers. Development committees, defined as health facility workers and representatives of the community regular meetings to discuss issues and ways to improve healthcare provision and attendance (in one case specific drugs uptake), were the main component of six studies (Björkman and Svensson, 2009; Olayo *et al*., 2014; Cannon *et al*., 2017; Pol *et al*., 2017; Oguntunde *et al*., 2018; Ndaba *et al*., 2020), two of which included also the involvement of community volunteers (Olayo *et al*., 2014; Cannon *et al*., 2017). The main protagonists of the other eight studies, of which five included a specific health facility strengthening component (e.g. staff training, equipment and drugs provision) (Bari *et al*., 2006; Waiswa *et al*., 2015; Gupta *et al*., 2017; Awasthi *et al*., 2019; Var *et al*., 2020), were health volunteers, who create a bridge between the local community and facility by conducting health education, promotion, quality improvement processes (at community and/or household level) and providing Primary Health Care (Kamugisha *et al*., 2018; Tancred *et al*., 2018; Kushitor *et al*., 2019). Only one intervention included economic incentives for participants—cash benefit for mothers, with free delivery and free treatments for sick neonates (Gupta *et al*., 2017).

### Intervention effects on child health

All the papers highlighted positive effects resulting from the interventions. While a statistical meta-analysis was not conducted, the individual studies consistently reported favourable outcomes (Table 2).

**Table 2.** Studies’ key results

Reference	Key results	Comm. mobilization	Healthcare access changes	U5 mortality changes	CSB changes	HP changes	HF services/equipment changes	Relationship comm./HP changes
Oguntunde *et al*., 2018	FDCs and staff members: - FDCs can influence QoC (FSs provided, comm. mobilization) - costs can prevent successesClients: - low awareness of FDCs - FDC influence positively FS quality	For FDC: - 72.9% For clients: - 60.4%	For FDC members: - 49.4% For clients: - 57.6%			For FDC members: - increased availability (60.9%) - impr. attitude (60.2%) For clients: - 58.3% - 53.5%	Equipment availability impr.: - FDC (46.9%) - clients (35.4%) FSs provided expansion: - FDC (40.1%) - clients (38.5%) Reduced medicines stockouts: - FDC (35.6%) - clients (45.8%) Inadequate fundings - FDC (61.2%)	
Cannon *et al*., 2017	Intervention successful: - drugs’ use supported - cooperation and involvement of government, comm., local stakeholders, HPsChallenges: - stock outs, staff shortage - socio-cultural barriers (women cannot make decisions; myths/fears of medication)	Impr. awareness/access/utilization of the drugs		Estimated reduction of maternal/neonatal mortality (“saved the life of 40–50 women who would have died from PPH and 200 newborns from cord sepsis”)			New availability of drugs (but frequent stock outs)	
Awasthi *et al*., 2019	Increased care seeking for pneumonia from both HF and village-based interventions		Access to government HF: - from 5.4% to 17.9% in HF-based intervention - from 3.3% to 16.1% in village-based intervention		Csb impr.: - village level = 79.3% [95% CI (75.1–82.9)] (*P* < 0.0001) - HF level = 68.9% [95% CI (64.4–73.6)] (*P* = 0.02)			
Tancred *et al*., 2018	- Successful training of CHVs - Perceived impr. in hcare attendance - CSB shifted from traditional healers to HF - Men more sensitized towards maternal and newborn health - Impr. demand not met by increased healthcare system capacity	- Comm. men more involved in their wives and children’s health	Perceived increase in hcare attendance of comm. members		Perceived shift towards increases in HF csb		Perceived problems with increased hcare demand not met by an increased capacity of healthcare provision	
Kushitor *et al*., 2019	Positive ranking for CHPS (7.7/10): - appreciation, pride, satisfaction, confidence in CHPS FSsNegative aspects: - HP issues (poor human relations/extorsion) - health system (no essential logistics) - men felt excluded from FSs - FSs are not tailored to community needs		Increased access due to: - care impr. - reduced costs				Poor equipment and specialized FSs reported	Reported as poor due to rudeness of HP
Var *et al*., 2020	Intervention vs control: - HPs: impr. in danger signs recognition, hygiene practices - VHSG: impr. in danger signs recognition, visits to children and hygiene knowledge - mothers: impr. knowledge but no change in csb, referral, hygiene	- Mothers’ knowledge of newborn danger signs impr. [OR 2.35 (1.22–4.52); *P* = 0.0104] - Mother’s hygiene measures no significant change [OR 1.62 (0.81–3.22); *P* = 0.1697]	Referral time no impr. [OR 0.45 (0.19–1.06); *P* = 0.0688]		No change	- Hygiene practices impr. [OR 15.60 (7.73–31.47); *P* < 0.0001] - Danger signs knowledge impr. (OR 35.91 (19.31–66.78); *P* < 0.0001		
Olayo *et al*., 2014	Impr. in: - antenatal care - HF delivery - water and sanitation use - insecticide-treated nets use - presence of clinic card - measles vaccination	- Water use [OR 5.12 (4.87–5.383); *P*≤0.001] - ITN [OR 1.115 (0.881–1.410); *P* = 0.3666] - Food [OR 2.679 (2.56–2.803); *P≤*0.001]	- Clinic card [OR 0.439 (0.302–0.638); *P*≤0.001] - Measles vaccination [OR 1.144 (1.011–1.296); *P* = 0.0335]					
Kamugisha *et al*., 2018	- Households from hard-to-reach areas had less HF deliveries - Nurse led visit to the neonate associated with more complete immunizations and infant survival rates - No change in nutrition status		- Less in hard-to-reach households compared to other [OR 0.55 (CI 0.45–0.67); *P *≤ 0.001] - Increased immunization rates in households that received a nurse led visit [OR 1.64 (CI. 1.19–2.27); *P* = 0.002]	Infant survival at 12 months: - better if had a nurse-led visit [OR 1.89 (CI 0.99–3.61); *P* = 0.05] - worse if being from a hard-to-reach area [OR 0.70 (CI 0.52–0.96); *P* = 0.03]				
Pol *et al*., 2017	- Perceived QoC increase at the HF - Participants felt impr. in their health knowledge and care practices - HPs perceived their care provision impr. thanks to knowing better the needs of the patients - Comm. members felt empowered and more responsible of their own health - Communication between HP and comm. members impr.	Impr. in: - health knowledge - care practices - participation in health activities				Impr. of care provision thanks to better understanding of comm. needs	QoC perceived as impr. by all participants	Impr. communication between comm. members and HPs
Gupta *et al*., 2017	- Immunization rates increased for girls - HF impr. (equipment, sanitation, drugs availability, but not staff) - Increased healthcare access		- Increased utilization of ambulances and HFs thanks to SHA - Disparity reduction in immunization rates (still mistrust, girls exposed to vaccines only because considered more expendable than boys)		impr. thanks to the SHA	Inadequate staff increase to cover all the new programmes	- Increased number and availability of equipment at HFs, but not as much as expected - Increased drugs availability and referral system (but no maintenance/repair of broken ambulances)	
Bari *et al*., 2006	Increases in: - self-referral of sick newborns - compliance after CHWs referral - care-seeking from qualified HPs and HFsDecreases in: - CSB from unqualified HPs	Increased self-referral of sick newborns (even if not assessed as sick by CHWs) (48.1% of newborns arriving to the hospital)	Changes in care sought from the hospital (int.vs.control):OR 2.90 (1.91–4.41); (*P* < 0.0001)		Csb from: - qualified HP (int.vs.control):OR 2.98 (2.00–4.44); (*P* < 0.0001) - unqualified HP (int.vs.control):OR 0.31 (0.21–0.47); (*P* < 0.0001)			
Waiswa *et al*., 2015	Increases in: - immediate/exclusive breastfeeding - umbilical cord cutting with a clean instrument/ dry cord care - bathing the newborn after 24 hNo changes in CSB (already high)Home-visits by CHW more to women who delivered at home rather than at HF	Int. vs control: - impr. immediate (72.6% vs 66.0%; *P* = 0.0116) exclusive (81.8% vs 75.9%; *P* = 0.042) breastfeeding - first bath >24 h (49.6% vs 35.5%; *P* < 0.001) - dry cord care (63.9% vs 53.1%; *P* = 0.002) - kangaroo care (22.4% vs 9.3%; *P* = 0.089)	- Impr. use of HFs and skilled birth attendance (both intervention (21.3% more) and control (19.2% more) arm) - Decrease in private HFs attendance (13% less) (control arm—2.5% less)	study not powered to detect differences	increased csb for both int. and control		- Impr. equipment - More than 60% facilities reported at least one stock-out	
Ndaba *et al*., 2020	- Created an environment for communication and engagement - Identified PHC weaknesses/threats/risks/strengths/opportunities - Improved care of pregnant mothers and their babies pre- and post-delivery	- Increased engagement of comm. members on health matters - Earlier referral to PHC facilities of neonates with abnormalsigns and symptoms			Reduced home deliveries (ambulances called as soon as possible when mothers go into labour)			Impr. communication between comm. members and HPs
Björkman and Svensson, 2009	- High comm. engagement - Positive changes in the facilities and in the HPs - Higher FS utilization - Higher infant weight - Reduction of under-5 mortality	Increased participation of comm. members	16% higher utilization (outpatient services) compared to control group	1.7% less child deaths compared to control group		19% reduction of absence rates compared to control group	- Presence of suggestion boxes - Utilization of waiting cards and reduction of waiting time - Correct equipment usage - Better conditions of the clinics	

Abbreviations: comm. = community; CHV = Community Health Volunteer; CHW = Community Health Worker; csb = care-seeking behaviour; DC = Development Committee; FGD = Focus Group Discussion; FS = Facility Services; HF = Health Facility; HP = Healthcare Providers; impr. = improvement; int. = intervention; QoC = Quality of Care.

#### Care-seeking behaviour

Changes in care-seeking behaviour were measured in by seven studies (Bari *et al*., 2006; Waiswa *et al*., 2015; Gupta *et al*., 2017; Tancred *et al*., 2018; Awasthi *et al*., 2019; Ndaba *et al*., 2020; Var *et al*., 2020). Of them, only one reported no change (Var *et al*., 2020). Among the positive results, one highlighted this change both in the intervention and control groups (Waiswa *et al*., 2015), and another reported an increase in care-seeking from qualified providers for sick new-borns [(int.vs control): OR 2.98 (2.00–4.44); *P* < 0.0001] together with a decrease from unqualified providers in comparison to the control arm [(int. vs control): OR 0.31 (0.21–0.47); *P* < 0.0001] (Bari *et al*., 2006).

#### Healthcare access

Eleven of the 14 papers measured changes in community members’ healthcare access (Bari *et al*., 2006; Björkman and Svensson, 2009; Olayo *et al*., 2014; Waiswa *et al*., 2015; Gupta *et al*., 2017; Kamugisha *et al*., 2018; Oguntunde *et al*., 2018; Tancred *et al*., 2018; Awasthi *et al*., 2019; Kushitor *et al*., 2019; Var *et al*., 2020), with 8/11 reporting an increase in access. One study not only reported a rise, although not significant, in public health facilities attendance for both intervention (21.3% increase) and control (19.2%) groups, but also documented a decrease in private facilities visits only in the intervention arm (13% reduction, opposed to 2.5% in the control) (Waiswa *et al*., 2015). Another study reported hard-to-reach households had less improvement in this outcome, compared to less isolated ones [OR 0.55 (95%CI 0.45–0.67); *P* ≤ 0.001] (Kamugisha *et al*., 2018). Var *et al*., instead, reported an improvement in referral time, although was not statistically significant [OR 0.45 (0.19–1.06); *P* = 0.0688] (Var *et al*., 2020). Improvements in healthcare access were measured through healthcare facility and emergency transport utilization, use of clinic cards, but mostly via an increase in immunization rates. In one study, a reduction of gender disparity in vaccine uptake to the point of having more girls vaccinated than boys was reported. However, the complementary qualitative analysis revealed mistrust towards this practice despite the intervention, with girls being vaccinated because they were considered more expendable than boys by their parents, fearing vaccines’ negative events (Gupta *et al*., 2017).

#### Community mobilization

Ten studies assessed community mobilization (Bari *et al*., 2006; Björkman and Svensson, 2009; Olayo *et al*., 2014; Waiswa *et al*., 2015; Cannon *et al*., 2017; Pol *et al*., 2017; Oguntunde *et al*., 2018; Tancred *et al*., 2018; Ndaba *et al*., 2020; Var *et al*., 2020). This outcome refers to improvements in health knowledge and practices (including vaccination uptake), changes in hygiene measures and water and sanitation practices in households, participation in health activities and self-referral of sick children. Improvements were reported by all studies, with only one assessed outcome—hygiene measures, not significantly increased in one study (Var *et al*., 2020), and another study reporting socio-cultural barriers, despite a successful intervention (Cannon *et al*., 2017). While most of the studies focused exclusively on mothers, one reported a rise in involvement of men in child health (Tancred *et al*., 2018).

#### Healthcare facilities’ services provision

Changes in facility services provision were mentioned by nine papers (Björkman and Svensson, 2009; Waiswa *et al*., 2015; Cannon *et al*., 2017; Gupta *et al*., 2017; Pol *et al*., 2017; Oguntunde *et al*., 2018; Tancred *et al*., 2018; Kushitor *et al*., 2019). Improvements in quality of care, equipment, waiting times, drug availability and referral systems were reported by most of them. They were often matched, though, with reports of inadequate funding, which would lead to compromises and only partial ameliorations. For example, Oguntunde et al.’s study reported committee members paying out-of-pocket for facility equipment (Oguntunde *et al*., 2018), Gupta et al. described how no maintenance plan/funds for the new referral system impeded broken ambulances repair (Gupta *et al*., 2017), and Waiswa et al.’s paper recounted that more than 60% of the facilities reported at least one drug stock-out (Waiswa *et al*., 2015). Moreover, one study pointed out poor equipment and supplies, with services provided not tailored to local community needs (Kushitor *et al*., 2019), and another reported that the increased demand gained was not met with an increase in health services capacity, resulting in inadequate patient assistance (Tancred *et al*., 2018).

#### Healthcare providers’ practices and relationships with community members

Changes related to healthcare providers were presented in six papers (Björkman and Svensson, 2009; Gupta *et al*., 2017; Pol *et al*., 2017; Oguntunde *et al*., 2018; Var *et al*., 2020), with overall positive outcomes. Improvements ranged from practices (hygiene) to expertise (knowledge and ability to recognize new-born danger signs), from availability and attitude towards the patients to a reduction of absenteeism from the facilities to overall care provision. Gupta et al.’s study reported an inadequate staff increase to meet all the new activities scheduled for implementation (Gupta *et al*., 2017). Pol et al.’s study, instead, explored the relationship with communities as another aspect of provider change (Pol *et al*., 2017). Healthcare workers recounted in their feedback reflections a better understanding of community needs (possible thanks to the establishment of a communication channel with the linkage intervention).

The relationship between community members and healthcare providers was qualitatively assessed by two other papers. While Ndaba et al. described improved communication and an overall stronger connection (Ndaba *et al*., 2020), Kushitor et al. reported a poor relationship, with community members complaining about the rudeness of the healthcare workers during Focus Group Discussions. In addition to this, men from the same study felt excluded from service provision, due to the focus exclusively on mothers and children (Kushitor *et al*., 2019).

#### Under-five mortality

Information on changes in mortality rates among children under the age of five was provided by three papers (Björkman and Svensson, 2009; Cannon *et al*., 2017; Kamugisha *et al*., 2018), with Waiswa et al. reporting that their study was not powered to detect differences in mortality between intervention and control (Waiswa *et al*., 2015). Cannon et al.’s qualitative case study reported initial estimates on undisclosed data by the programme’s staff that ‘[the medication object of the intervention] saved 200 newborns (who would have died) from cord sepsis’ (Cannon *et al*., 2017). Kamugisha et al. mentioned infant survival at 12 months, with odds of survival increasing if the neonate received a nurse-led home visit [OR 1.89 (CI 0.99–3.61); *P* = 0.05], and decreasing if the child lived in a hard-to-reach area [OR 0.70 (CI 0.52–0.96); *P* = 0.03] (Kamugisha *et al*., 2018).

### Community participation assessment

**Figure 2.**
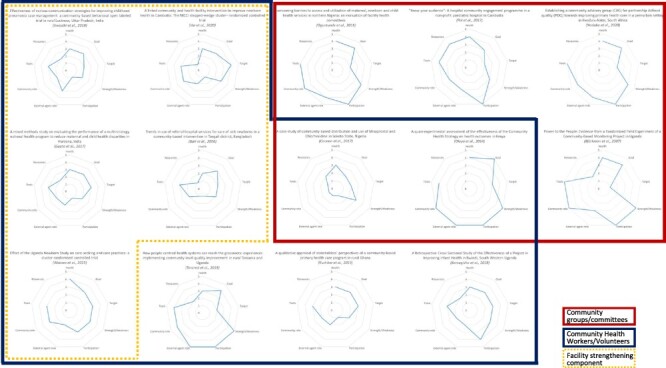
Spider diagrams to describe community participation

The included papers were assessed using Rosato’s framework, and a graphic representation of this classification can be found in Figure 2. We grouped the spider diagrams according to the type of intervention implemented, using this framework to offer a perspective to explain the outcomes of each of them. Overall, the variables’ scores provide an insight into which characteristics of the included studies carry the biggest transformative and community-empowering capacities and which areas need strengthening, to inform future interventions.

Of the nine variables, ‘Conceptualization of health’, ‘Goal’ and ‘Tools and Methods’ were the domains with overall good scores, while ‘Resources’, where applicable (five papers did not specify how funding was mobilized), was assigned low scores with the exception of one study (Pol *et al*., 2017). ‘Participation’ was the variable with the biggest variation, being the one with the highest number of papers scoring I (*n* = 6), as well as the highest number of papers scoring V (*n* = 6).

Five of the six studies that used community groups (two of which also involved community volunteers), had the overall highest scores, with ‘Participation’ scoring V in all of them. This is not surprising for Pol et al.’s paper, as community empowerment was the main intervention focus (Pol *et al*., 2017), nor for Olayo et al. who, by reporting participation as the most improved outcome, confirmed the importance of involving communities to obtain strong commitment to an intervention (Olayo *et al*., 2014). The spider diagrams of Oguntunde et al. and Ndaba et al.*’*s studies, instead, highlight that the lack of involvement of marginalized/at risk groups (‘Target’ score II), even with a high ‘Participation’ score, can lead to low awareness of the intervention in the community (Oguntunde *et al*., 2018) or to limited ability to reach isolated parties (Ndaba *et al*., 2020). Some of Cannon et al. case-study’s low scores (‘Participation’, ‘Community Role’ and ‘Tools’ I), then, explain why the intervention did not succeed in tackling local socio-economical barriers, changes in which were reported as something needed to strengthen the effectiveness of the implementation (Cannon *et al*., 2017).

Community volunteers/healthcare workers were the core of most interventions, with five of them including a health facility strengthening component. The scores overall were lower, with a few exceptions, like Tancred et al.’s study: the high general scores confirmed the importance of empowering the community for a successful implementation, but at the same time low scores in variables like ‘Resources’ highlight that such empowerment needs to go hand in hand with a financial plan to improve and sustain the changes, explaining the inadequate service provision reported in the article (Tancred *et al*., 2018). Among these papers, ‘Participation’, ‘Community role’ and ‘External agent role’ scores were generally low, which could help to explain the poor caregivers’ care seeking behaviour change outcomes of Var et al.’s paper (probably due to a lack of engagement of community members in the intervention design and implementation) (Var *et al*., 2020), or the mistrust towards immunization reported by Gupta et al. (Gupta *et al*., 2017), confirming once more how interventions should focus on empowering community members and make them protagonists, not just recipients of health implementations.

### Realist assessment

Following realist principles, we extracted the information on the programme theories behind the interventions and developed CMO configurations to deduce the MRTs from each paper (Table 3). By doing so, we noticed that the included studies had many similarities.

**Table 3.** Studies’ CMO configuration

			Mechanism			
Reference	Programme theory	Context	Resources	Reasoning	Outcome	Middle-range theory
Oguntunde *et al*., 2018	Establishing local health committees can improve QoC and attendance to services	- No DC present before in the communities - Rural context - Low trust towards hospitals/low attendance	Institutions of health committees (unpaid) with members selected by the comm. among themselves	Participants were active in the committee, and health staff was responsive to interactions with them	Improved healthcare delivery, and strengthened connection between hospitals and comm.	DCs as a bridge between comm. and facility can work nicely as long as the members are people trusted in the comm.
Cannon *et al*., 2017	Comm. provision by non-skilled attendants of drugs to prevent post-partum haemorrhage and neonatal cord sepsis can reduce maternal and neonatal deaths	- Rural context- Low facility attendance - Presence of Ward DCs - Presence of CHVs - Presence of drug keepers (pharmacists)	- Drugs procurement and distribution - Training of HPs (in the facilities and CHV) and drug keepers on the use of these drugs	- Most participants (mothers/husbands/healthcare providers) responsive to the int., appreciating drugs’ benefits - Comm. engaged and participating in awareness/usage of drugs - Some comm. members still sceptical, mistrust of the drugs	Maternal and newborn deaths estimated to have reduced, despite frequent stock outs and still some mistrust towards them	Comm. distribution of drugs requires reliable drug stocks, transportation and comm. trust in order to work to reduce maternal and neonatal mortality
Awasthi *et al*., 2019	Infrastructure strengthening plus facility and comm.-based behaviour change int.—using education, information, and communication materials—can improve csb for pneumonia from the public healthcare system	- Mistrust towards public healthcare systems - Rural area	- Impr. of infrastructures and equipment - Re-training of staff in the health facilities - Presence of Social Health Activists and Auxiliary Nurses and Midwives responsible for comm. health	- Comm. members participated actively in the awareness sessions, even expressing interest in spreading the information received among their peers - Healthcare providers were responsive to training and helped to facilitate the meetings	Increased csb to government facilities for pneumonia-related symptoms (more when awareness sessions were held at village-level)	Facility + comm. int. need healthcare providers’ strong commitment and comm. members’ responsiveness to improve csb
Tancred *et al*., 2018	Quality impr. using PDSA cycle can be done simultaneously at comm., facility and district level	- Rural contexts (both Uganda and Tanzania) - Pre-existing government employees took the role of monitoring comm. groups	Establishment of CHW among comm. members (chosen by themselves based on determined categories)	- CHWs were very active, even if struggling with concepts of comm. participation or collecting data - HPs appreciated the CHWs efforts - Comm. leaders were very responsive - Men were very responsive	Impr. in comm. ability to deal with health-related issues, and HPs	Quality impr. requires collaborative action at both comm. and facility levels to succeed and bring societal changes
Kushitor *et al*., 2019	Project introducing Comm. Health Officers to be based in the comm. providing PHC can empower rural communities in the governance of their own healthcare services	- Rural area - Poor healthcare provision - Patriarchal society with men controlling resources	- Establishment of Comm. Health Officers (CHO) - Establishment of Comm. Health Compounds (CHC)—health posts for PHC provision	Comm. members were appreciative of the idea, but complained about the actual implementation (especially men and young boys who felt excluded)	While the int. concept was well-received, it was poorly implemented, recording no impr. in the relationship with HPs, services not tailored for the comm. needs, comm. members not engaged (especially men)	To empower local communities, all comm. members need be included and participate in the planning, budgeting, implementation phases of the project
Var *et al*., 2020	Coordinated int. (HP training + CHWs home visits for cases early detection + improved caregivers’ health knowledge) can improve neonatal health and csb	- Presence of village DCs - Recipients of previous successful int. to reduce U5 mortality (but did not work on neonatal deaths) - Rural area	Funds allocated to all levels of int., equipment provision, training	Caregivers improved their knowledge and changed hygiene habits, but their csb remained unchanged as external conditions did not improve	Impr. in all three cadres in knowledge and hygiene practices, but not in cs behaviour	In order to change behaviours as well as knowledge, multi-level approaches need to consider and change all the external factors that influence actions (i.e. roads security)
Olayo *et al*., 2014	Comm. participation can improve health outcomes also when implemented in different socio-economic contexts	- Rural, peri-urban and nomadic areas - CHWs and DCs already established in the communities	- Reinforcement of committees at comm. and facility level - Training of HCW	Participants changed their attitudes around many health-related issues after receiving education on these matters	Impr. in all the health behaviours in all settings, with greater changes in the rural one	Resources allocation and service deliveries need to be adjusted to the different settings based on needs for comm. participation activities to succeed in different socio-economic contexts
Kamugisha *et al*., 2018	Nurses and CHVs-led comm. health program can help reduce infant mortality and improve deliveries at health facilities, immunization rates and children’s nutritional statuses	- Rural area - Low attendance to healthcare facilities - Poor road network	Recruitment and training of comm. nurses and CHVs	Almost all mothers were responsive to visits from comm. nurses and CHVs and improved their health-related behaviours	Reduction in infant mortality rates and impr. in immunization completion rates, deliveries at health facilities (less with babies from hard-to-reach areas). No change on nutritional status (but <2% of infants reported as malnourished from the beginning)	Multi-sites comm. health programmes need to be more inclusive (i.e. tailor solutions for hard-to-reach areas) to obtain equal impr. in different int. areas
Pol *et al*., 2017	Comm. engagement, health education and relationship strengthening with healthcare professional improve QoC and can benefit from meeting set in informal venues (i.e. cafes)	- Rural area - Free healthcare for <16 children - Interest in providing high QoC (HPs) - Social norms recommend not questioning who is in a position of authority	- Hospital staff member implemented, organized and facilitated two groups of comm. engagement	Participants were very receptive and showed enthusiasm, to the point of asking more meetings	Impr. in QoC, service delivery, communication between comm. and HPs, health knowledge and comm.’s sense of empowerment	Comm. engagement activities promoted in informal venues improve QoC and facilitate participants’ empowerment and participation in health matters
Gupta *et al*., 2017	Implementing a program that offers free health services (medicines, transport, visits, treatments) and home-visits by local CHV can reduce maternal and child health inequalities	- High MCH inequalities (rural vs urban, poor vs rich) - High socio-economic inequalities among and within state districts - Women: low literacy, lower status in society - Lack of political will	- Increased number of health centres - Recruitment and training of CHVs - Free multiple health services and transport provision	Participants were very responsive to the SHAs, increased trust	- Overall reduction in MCH inequalities and impr. in MCH outcomes (but goal not reached) - Disparity in vaccination uptake between boys and girls - Service provision better in some areas than others	To reduce inequalities, services provision needs to be uniformed to each setting needs and the socio-economic-political issues of participants need to be addressed (i.e. women empowerment)
Bari *et al*., 2006	Given that quick referral/assistance can decrease neonatal mortality, CHWs’ programmed home visits can detect early if urgent medical care for newborns is needed and can speed up the referral process	- Low awareness of signs and symptoms of neonatal distress in the comm. - Various social factors can delay referral to the hospital (from need to have father’s consent, to mother and child having to be confined for a few weeks after delivery) - Rural context	- Training of HCWs - Adaptation during the int. to include use of digital weight scales - Health education to families during pregnancy	Receiving health education helped the families to seek adequate care from hospitals (sometimes even when advised against by CHWs)	Increased care-seeking rates, both from referral by HCWs and from self-referral	In a context where awareness on neonatal danger signs among caregivers is very low, health education and awareness creation can decrease neonatal mortality
Waiswa *et al*., 2015	CHWs can improve neonatal mortality and newborn care practices in SSA (as it did in South Asia) by doing frequent home visits to new mothers	- Comm. component of the health system already existing and working well, decision to expand the program with a focus on newborn care - Rural context	- Collaboration of the local government, approval of district authorities and local leaders - Training of HCWs by experts	Comm. members chose the HCWs to be someone they trusted and can accept in their homes	Increased public facilities utilization and better healthcare practices	When comm. health is already accepted in the setting, new components (i.e. neonatal care) can be easily added, and the possibility to choose their own HCWs facilitates the process
Ndaba *et al*., 2020	Establishing Comm. Advisory Group can improve the relationship between health facilities and local communities and therefore improve the quality of healthcare services	- Poor MCH care at facility and comm. level - Poor linkage between communities and facilities - Pre-existing successful program linking communities and government- Socio-political stability	Institution of comm. advisory groups with members selected among comm. members, healthcare providers, faith organizations and NGOs	Participants were active in the group meetings and prone to make changes	Improved: - communication and engagement among MCH stakeholders - healthcare attendance - PHC services provision to mothers and children	CAG can improve QoC and link communities and facilities as long as local ownership is ensured, and can benefit from political stability and previous comm. structures to collaborate with
Björkman and Svensson, 2009	Rural communities can be able to hold primary healthcare providers accountable and lead to improved healthcare delivery	- Weak primary health care delivery - Rural setting - Poor comm.’s health knowledge and awareness of the status of services delivery	Institution of citizen reports cards and utilization of participatory methodology to engage comm. members in health-related matters	Active participation of comm. members, with increased awareness of health issues related to their local facilities and solution-finding	- Impr. quality and quantity of primary healthcare provision - Impr. health outcomes - Behaviour change of HP - Improved comm. engagement	Rural communities, given the right tools and knowledge, can improve healthcare delivery, as long as there is participation and engagement of HP as well

Abbreviations: comm. = community; CHV = Community Health Volunteer; csb = care-seeking behaviour; DC = Development Committee; HP = Healthcare Providers; impr. = improvement; int. = intervention; MCH = maternal and child health; QoC = Quality of Care.

The programme theories behind each intervention, for example, followed the same general principles: community participation/education, communication with healthcare providers (via meetings, or the establishment of intermediate bodies like committees), and health services ameliorations, can all lead to improvements in quality of care, health outcomes and/or relationship between communities and healthcare providers.

Context and Resources varied based on the different specifics of the setting and the intervention, despite all being set in rural LMIC areas. However, the various participants were in general very responsive to these types of interventions (Reasoning), even when issues were reported with the actual implementation (compared to the original idea) and the exclusion of some populations such as men (Kushitor *et al*., 2019), or when participants were not able to act upon what they learned during the intervention due to unchanged external matters (Var *et al*., 2020).

**Figure 3.**
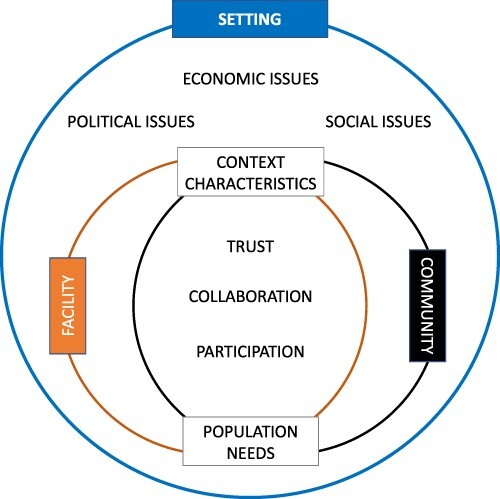
New framework based on studies MRTs

The MRT that we obtained from each paper showed some key factors in common. As MRTs can function as starting points for future intervention designs, we summarized the shared elements that the MRTs showed and built a new framework to inform future implementation and analysis plans (Figure 3).

Trust, collaborative spirit and active participation emerged as fundamental aspects for an intervention like community–facility linkage to succeed (which are also among the attributes in Rosato’s framework). While one could consider the importance of these attributes only among community participants, lessons learned from these papers show how valuable it is for these same characteristics to be present in healthcare providers as well. Other important factors for successful interventions pertain to the intervention setting in which the community of interest lives and acts: socio-economic dynamics, including gender disparities, power distribution/political hierarchies, external factors like transportation means/road security to materially link the community and its local health facility, any of these factors can influence these intervention’s outcomes and must be considered in future implementations. In light of these reflections, it becomes clear how tailoring the implementation to the actual characteristics of the context and the needs of the recipient community is vital to obtain equality and sustainable transformation over time—echoing the calls from the literature for inclusion of local communities’ voices in every step of development interventions as a way to achieve long-lasting improvements (Campbell and Jovchelovitch, 2000; Andersson, 2011; Petiwala *et al*., 2021).

## Discussion

In this review, we assessed past interventions linking communities and local facilities to understand which features can influence their successes. Overall, all the studies reported positive results, from improved care-seeking behaviour and healthcare access for community members, to increased expertise and better practices for healthcare providers. Community mobilization, measured in terms of discrete health practices rather than wider shifts in norms, showed positive changes but proved that some of these interventions’ designs were incapable of tackling socio-economic barriers. Health facilities’ service provision improved generally, but some issues were flagged, from inadequate funding to sustain such changes to new services being offered that did not adapt to the local needs. Finally, one peculiar finding was that impacts on child mortality rates were not properly assessed by any of the papers.

In terms of design and delivery conditioning aspects, lack of awareness of the intervention among local community members often led to only partial achievement of changes. For this type of intervention to be effective, active participation and engagement should be put in place, as without high community involvement long-lasting and effective improvements are unlikely to be obtained. Moreover, hard-to-reach households need to be taken into account more carefully. The authors of papers in which the exclusion (even if involuntary) of hard-to-reach communities led to only partial success of the projects stressed the need to increase efforts to include entire populations more actively in future implementations, to obtain sustainable and equitable changes. This point feeds into a bigger discussion on the need to include equity impact routinely in any study design and implementation; only when it becomes the norm, the risks of unintended effects like the ones reported here start to reduce (Morrison *et al*., 2019; Spencer *et al*., 2019). When considering the need to be as inclusive as possible, then, involvement of fathers in a type of intervention generally delivered to the children’s mothers becomes another key point (Tully *et al*., 2017). As they are usually the heads of the family, responsible for any financial and health-related decision, and there is evidence that their presence in such studies can improve child health, it would be advisable to routinely include them in any implementation. When fathers have been included, their interest and involvement in child health have increased (Tancred *et al*., 2018). Moreover, their exclusion could potentially harm the attendance to care of women and children, given the aforementioned decision-making role of men in the family (Kushitor *et al*., 2019).

Another point of discussion is the importance to match health interventions with financial plans, as a lack of funding can hinder the sustainability of the changes, and one could incur in the risk to be unable to meet a demand increase (i.e. vaccines requests after an education programme) with an adequate supply provision (sufficient number of vaccine doses in the local facilities). Since a failure to satisfy these standards would undermine the whole purpose of a linkage intervention, it becomes clear the need to work on all sides of an implementation.

A further issue highlighted by our analysis of Kushitor et al. (Kushitor *et al*., 2019) is the challenge of maintaining the same structure for the intervention over time. A programme that was supposed to be carried over by the community became exclusively a facility intervention—with community engagement activities reducing during the course of the implementation. The loss over time of the community empowerment and engagement component might be explained by some features of the intervention design, which showed poor ownership of the programme [see ‘Strength/weaknesses’ (II), ‘Participation’ (I), ‘Community role’ (II) in Figure 2]. This emphasizes the importance of including sustainability from the start, achievable through early involvement of community voices, to be able to carry it over time.

For what concerns outcomes of interest, then, it was fascinating to observe that changes in mortality rates are practically not used to measure changes obtained by this type of intervention. Determining differences in such variable, though, could be extremely impactful, especially when considering implementations in areas with high child mortality rates. It might be worth exploring the feasibility of including it among the indicators of future implementations, whilst being conscious of the challenges it would entail—i.e. larger samples needed.

In terms of study methodology, we believe that to better understand the real effect of this type of intervention, mixed methods approaches are desirable. Outcomes like the increase in immunization rates for girls in Gupta et al.’s paper would have just appeared positive with an exclusive quantitative approach (Gupta *et al*., 2017). The qualitative data collected allowed the authors to understand that the health education component was not as successful, with the local population still afraid of vaccines. Plus, it helped to unveil gender disparity issues, awareness of which could inform future implementations. A thorough formative work with communities could have probably detected (and better addressed in the implementation phase) this issue, confirming once more the importance of carefully studying and including local populations’ voices in the design and delivery of an intervention (Trickett *et al*., 2011).

Some limitations must be acknowledged in relation to the studies included in this review. In regard to the data collection process, a few studies acknowledged the possibility of recall and selection bias (Waiswa *et al*., 2015; Cannon *et al*., 2017; Pol *et al*., 2017; Var *et al*., 2020), some potential issues with the quality of data (i.e. self-reported) (Cannon *et al*., 2017; Kamugisha *et al*., 2018; Kushitor *et al*., 2019; Var *et al*., 2020) and with confounding factors (other development projects undergoing in the same study area) (Olayo *et al*., 2014; Gupta *et al*., 2017). For what concerns the data analysis, some papers recognized the lack of control groups (Gupta *et al*., 2017; Kamugisha *et al*., 2018; Oguntunde *et al*., 2018), small sample size (Waiswa *et al*., 2015; Tancred *et al*., 2018; Awasthi *et al*., 2019; Ndaba *et al*., 2020; Var *et al*., 2020) and early stage analysis (i.e. before study completion) (Björkman and Svensson, 2009; Pol *et al*., 2017; Ndaba *et al*., 2020) as limitations to their studies.

With respect to our application of Rosato’s framework, it is not surprising that the variables of Health, Goal and Tools have generally good scores in this type of intervention. ‘Conceptualization of health’ as more than the absence of disease and disorders (score I: Medical model); ‘Goal’ including health knowledge and behaviour change as well as the eradication of specific health problems; and ‘Tools’ including behaviour-change and social capacity-building methods rather than simply clinical ones. All these are prerequisites of a community-facility linkage intervention. For variables like ‘Participation’, ‘External agent role’ and ‘Community role’, it was in general more challenging to attribute the highest scores. Leaving the intervention decision-making process entirely in the hands of an auto-sufficient community functioning as the main active agent of change, with the external representatives acting only as an additional support resource, is the most challenging part to achieve. Analysing the papers via this framework, though, has given more evidence that when this effort is made (and community is really empowered and is the active protagonist of the intervention), better outcomes are achieved. That said, it must be noted that scoring the interventions based only on the condensed study description sections—and on the reviewers’ subjective interpretations—could not render justice to them. One way to improve the reliability of the attributed scores would be to contact the study authors and have them grade their own work according to the integrated framework’s guidelines—and compare the results. In any case, these scores are not to be interpreted in absolute terms, but just as a useful tool to guide the analysis of the community participation component of these studies, and to better understand what is needed for an intervention to become truly empowering and sustainable.

We used realist principles for a review of the literature, but we did not follow the meticulous structure of Realist synthesis (Rycroft-Malone *et al*., 2012). We appreciate that this could be perceived as unconventional. We agreed on this hybrid structure because we wanted to incorporate the explanatory aims of realist principles into the systematic review’s evaluative nature, as we believed it could help to answer our research question in a more complete way. During the inception phase of the study, we decided to limit the search to academic publications, aiming to uphold the quality, reliability and rigour of our systematic review. However, we recognize that this choice does come with restrictions, and we acknowledge that there is a limitation in our choice to restrict our focus solely to peer-reviewed articles from academic databases. This is not advisable when performing a realist synthesis, which encourages the exploration of a broader range of sources, including reports and various forms of literature.

In addition to this, we note that our decision not to use checklists for risk of bias assessment could present a limitation to our study as it may have resulted in us not checking all aspects that may have led to bias in the included studies. However, we are confident in our ability to critically assess the papers included in the review, given our extensive experience with systematic and peer reviews.

One further limitation is associated with our decision to include only studies written in English, primarily due to restricted translation capabilities, which could potentially introduce language bias into our findings. However, it is worth highlighting that we did not encounter any non-English publications during our search.

Our analysis of factors that contributed to the success or limitations of these studies has highlighted the following key lessons: the need for more active inclusion of hard-to-reach populations, preference for mixed-methods methodology, involvement of community members in programmatic decisions and a focus on the actual context need. This illuminates the importance of transformative considerations within evaluation plans, and Figure 3 suggests a potential evaluation framework that can be used to assess the impact of community-based and facility link interventions. This review offered an opportunity to reflect on this type of intervention, which, despite its potential being recognized at the international level, is still not particularly diffused. We provided an angle to analyse it, including a community empowerment integrated framework and application of realist principles, all trying to shed light on key features for the success of community-facility linkage interventions. We hope this will be helpful to influence other implementations and serve as a guide for future evaluation plans.

## Data Availability

The data underlying this article are available in the article and in its online supplementary material.

## Reflexivity statement

The author team represents diverse professional backgrounds with a range of research experience and expertise. The group includes researchers from three different countries (UK, Nigeria and Sweden). Five authors are female and three authors are male. This study contributes to A.I.’s PhD study through University College London. A.I., S.F. and A.A.B. are PhD students. P.V. and I.H. are experienced in designing and delivering development projects. R.B., C.K. and T.C. are senior global health academics and expert researchers.

## Appendix 1: Search strategy

### Pubmed

– 240 results
– Search strategy:

(((health facilities/or exp hospital units or exp hospitals/) OR ((health facility* or hospital*).mp)) AND ((community health workers/) OR ((community based organisation* or community based organization* or CBO* or community development organisation* or community development organization* or CDO* or civil society organisation* or civil society organization* or CSO* or community health worker*).mp.))) AND ((exp child/ or paediatrics/) OR ((child* or schoolchild* or paediatric* or paediatric*).mp.))

## Web of Science

– 975 results
– Search strategy:

1: ALL = (health facilit*)

2: ALL = (hospital unit)

3: ALL = (hospital*)

4: #1 OR #2 OR #3

5: ALL = ((community based organisation* or community based organization* or CBO* or community development organisation* or community development organization* or CDO* or civil society organisation* or civil society organization* or CSO* or community health worker*))

6: ALL = (community health workers)

7: #5 OR #6

8: ALL = (linkage or referral)

9: ALL = ((child* or schoolchild* or paediatric* or paediatric*))

10: ALL= (child or paediatrics)

11: #9 OR #10

12: #11 AND #7 AND #8 AND #4

## Embase

– 395 results
– Search strategy:

1. health facilities/ or exp hospital units/ or exp hospitals/

2. (health facility* or hospital*).mp.

3. 1 or 2

4. community health workers/

5. (community based organisation* or community based organization* or CBO* or community development organisation* or community development organization* or CDO* or civil society organisation* or civil society organization* or CSO* or community health worker*).mp.

6. 4 or 5

7. (linkage or referral).mp.

8. exp child/ or paediatrics/

9. (child* or schoolchild* or paediatric* or paediatric*).mp.

10. 8 or 9

11. 3 and 6 and 7 and 10

12. community participation/

13. (community participation or community engagement or community strategies).mp.

14. 12 or 13

15. 3 and 10 and 14

## References

[R1] Andersson N . 2011. Building the community voice into planning: 25 years of methods development in social audit. *BMC Health Services Research* 11: 1–17. 22376121 10.1186/1472-6963-11-S2-S1 PMC3397387

[R2] Awasthi S, Kumar D, Mishra N, Agarwal M, and Pandey CM. 2019. Effectiveness of various communication strategies for improving childhood pneumonia case management: a community based behavioral open labeled trial in rural Lucknow, Uttar Pradesh, India. *BMC Public Health* 19: 1–11. 31870334 10.1186/s12889-019-8050-0 PMC6929504

[R3] Bari S et al. 2006. Trends in use of referral hospital services for care of sick newborns in a community-based intervention in Tangail district, Bangladesh. *Journal of Health, Population and Nutrition* 24: 519–29. 17591349 PMC3001156

[R4] Bigdeli M, Rouffy B, Lane BD et al. 2020. Health systems governance: the missing links. *BMJ -Global Health* 5: e002533. 10.1136/bmjgh-2020-002533 PMC7422628 32784214

[R5] Björkman M, Svensson J. 2009. Power to the people: evidence from a randomized field experiment on community-based monitoring in Uganda. *The Quarterly Journal of Economics* 124: 735–69.

[R6] Campbell C, Jovchelovitch S. 2000. Health, community and development: towards a social psychology of participation. *Journal of Community & Applied Social Psychology* 10: 255–70.

[R7] Cannon M, Charyeva Z, Oguntunde O et al. 2017. A case study of community-based distribution and use of misoprostol and chlorhexidine in Sokoto State, Nigeria. *Global Public Health* 12: 1553–67. 27100376 10.1080/17441692.2016.1172102

[R8] Cha S, Jin Y. 2020. Have inequalities in all-cause and cause-specific child mortality between countries declined across the world? *International Journal for Equity in Health* 19: 1–13. 10.1186/s12939-019-1102-3 PMC6938619 31892330

[R9] Dalkin SM, Greenhalgh J, Jones D et al. 2015. What’s in a mechanism? Development of a key concept in realist evaluation. *Implementation Science* 10: 1–7. 25885787 10.1186/s13012-015-0237-x PMC4408605

[R10] Diaconu K, Falconer J, Vidal N et al. 2020. Understanding fragility: implications for global health research and practice. *Health Policy & Planning* 35: 235–43. 31821487 10.1093/heapol/czz142 PMC7050687

[R11] Gulaid LA . 2015. Community-facility Linkages to Support the Scale up of Lifelong Treatment for Pregnant and Breastfeeding Women Living with HIV: A Conceptual Framework, Compendium of Promising Practices and Key Operational Considerations.

[R12] Gupta M, Bosma H, Angeli F et al. 2017. A mixed methods study on evaluating the performance of a multi-strategy national health program to reduce maternal and child health disparities in Haryana, India. *Bmc Public Health* 17: 1–13. 28893214 10.1186/s12889-017-4706-9 PMC5594476

[R13] ISRCTN39213655 . 2019. The INSPIRING Project – building capacity to reduce child deaths in Jigawa State, Nigeria. 10.1186/ISRCTN39213655, Accessed 24 January 2022.

[R14] Iuliano A, Aranda Z, Colbourn T et al. 2020. The burden and risks of pediatric pneumonia in Nigeria: A desk‐based review of existing literature and data. *Pediatric Pulmonology* 55: S10–21. 31985170 10.1002/ppul.24626

[R15] Kamugisha SR, Dobson AE, Stewart AG et al. 2018. A retrospective cross sectional study of the effectiveness of a project in improving infant health in Bwindi, South Western Uganda. *Frontiers in Public Health* 6: 290. 10.3389/fpubh.2018.00290 PMC6194221 30370265

[R16] King C, Burgess RA, Bakare AA et al. 2021. Integrated Sustainable Childhood Pneumonia and Infectious Disease Reduction in Nigeria (INSPIRING) Through Whole System Strengthening in Jigawa, Nigeria: Study Protocol For a Cluster Randomised Controlled Trial. 10.1186/s13063-021-05859-5 PMC8802253 35101109

[R17] King C, Iuliano A, Burgess RA et al. 2020. A mixed‐methods evaluation of stakeholder perspectives on pediatric pneumonia in Nigeria—priorities, challenges, and champions. *Pediatric Pulmonology* 55: S25–33. 31985139 10.1002/ppul.24607

[R18] Kruk ME, Gage AD, Joseph NT et al. 2018. Mortality due to low-quality health systems in the universal health coverage era: a systematic analysis of amenable deaths in 137 countries. *The Lancet* 392: 2203–12. 10.1016/S0140-6736(18)31668-4 PMC6238021 30195398

[R19] Kushitor MK, Biney AA, Wright K et al. 2019. A qualitative appraisal of stakeholders’ perspectives of a community-based primary health care program in rural Ghana. *Bmc Health Services Research* 19: 1–13. 31533696 10.1186/s12913-019-4506-2 PMC6751899

[R20] McCoy DC, Hall JA, Ridge M. 2012. A systematic review of the literature for evidence on health facility committees in low-and middle-income countries. *Health Policy & Planning* 27: 449–66. 22155589 10.1093/heapol/czr077

[R21] Mertens DM . 1999. Inclusive evaluation: implications of transformative theory for evaluation. *American Journal of Evaluation* 20: 1–14.

[R22] Mertens DM . 2007. Transformative paradigm: mixed methods and social justice. *Journal of Mixed Methods Research* 1: 212–25.

[R23] Metwally AM, Abdel-Latif GA, Mohsen A et al. 2020. Strengths of community and health facilities based interventions in improving women and adolescents’ care seeking behaviors as approaches for reducing maternal mortality and improving birth outcome among low income communities of Egypt. *BMC Health Services Research* 20: 1–14. 10.1186/s12913-020-05412-1 PMC7322855 32600377

[R24] Morrison J, Osrin D, Alcock G et al. 2019. Exploring the equity impact of a maternal and newborn health intervention: a qualitative study of participatory women’s groups in rural South Asia and Africa. *International Journal for Equity in Health* 18: 1–12. 30971254 10.1186/s12939-019-0957-7 PMC6458781

[R25] Moucheraud C, Guo H, Macinko J. 2021. Trust in governments and health workers low globally, influencing attitudes toward health information, vaccines: study examines changes in public trust in governments, health workers, and attitudes toward vaccines. *Health Affairs* 40: 1215–24. 34339250 10.1377/hlthaff.2020.02006 PMC9060815

[R26] National Population Commission - NPC and ICF . 2019. Nigeria Demographic and Health Survey 2018 - Final Report. Abuja, Nigeria: NPC and ICF.

[R27] Ndaba T, Taylor M, Mabaso M. 2020. Establishing a community advisory group (CAG) for partnership defined quality (PDQ) towards improving primary health care in a peri-urban setting in KwaZulu-Natal, South Africa. *BMC Health Services Research* 20: 1–7. 10.1186/s12913-020-05275-6 PMC7206786 32384886

[R28] Oguntunde O, Surajo IM, Dauda DS et al. 2018. Overcoming barriers to access and utilization of maternal, newborn and child health services in northern Nigeria: an evaluation of facility health committees. *Bmc Health Services Research* 18: 1–11. 29426314 10.1186/s12913-018-2902-7 PMC5807838

[R29] Olayo R, Wafula C, Aseyo E et al. 2014. A quasi-experimental assessment of the effectiveness of the Community Health Strategy on health outcomes in Kenya. *Bmc Health Services Research* 14: 1–13. 25079378 10.1186/1472-6963-14-S1-S3 PMC4108865

[R30] Page MJ, Moher D, Bossuyt PM et al. 2021. The PRISMA 2020 statement: an updated guideline for reporting systematic reviews. *Bmj* 372: 1–9. 10.1136/bmj.n71 PMC8005924 33782057

[R31] Pawson R . 2013. *The Science of Evaluation: A Realist Manifesto*. SAGE Publications Ltd: Sage, 1–240.

[R32] Pawson R . 2017. Middle range theory and program theory evaluation: from provenance to Practice 1. In: *Mind the Gap*. New York: Routledge, 171–202 doi: 10.4324/9781315124537.

[R33] Pawson R, Greenhalgh T, Harvey G et al. 2005. Realist review-a new method of systematic review designed for complex policy interventions. *Journal of Health Services Research & Policy* 10: 21–34. 16053581 10.1258/1355819054308530

[R34] Petiwala A, Lanford D, Landers G et al. 2021. Community voice in cross-sector alignment: concepts and strategies from a scoping review of the health collaboration literature. *BMC Public Health* 21: 1–11. 33849498 10.1186/s12889-021-10741-9 PMC8042631

[R35] Pol S, Fox-Lewis S, Cheah PY et al. 2017. “Know your audience”: A hospital community engagement programme in a non-profit paediatric hospital in Cambodia. *PLoS One* 12: e0182573. 10.1371/journal.pone.0182573 PMC5542539 28771631

[R36] Rosato M . 2015. A framework and methodology for differentiating community intervention forms in global health. *Community Development Journal* 50: 244–63.

[R37] Rycroft-Malone J, McCormack B, Hutchinson AM et al. 2012. Realist synthesis: illustrating the method for implementation research. *Implementation Science* 7: 1–10. 10.1186/1748-5908-7-33 PMC3514310 22515663

[R38] Shittu F, Agwai IC, Falade AG et al. 2020. Health system challenges for improved childhood pneumonia case management in Lagos and Jigawa, Nigeria. *Pediatric Pulmonology* 55: S78–90. 31990146 10.1002/ppul.24660 PMC7977681

[R39] Spencer N, Raman S, O’Hare B et al. 2019. Addressing inequities in child health and development: towards social justice. *BMJ Paediatrics Open* 3: e000503. 10.1136/bmjpo-2019-000503 PMC6688679 31423469

[R40] Tancred T, Mandu R, Hanson C et al. 2018. How people-centred health systems can reach the grassroots: experiences implementing community-level quality improvement in rural Tanzania and Uganda. *Health Policy & Planning* 33: e1–e13. 29304250 10.1093/heapol/czu070

[R41] Trickett EJ, Beehler S, Deutsch C et al. 2011. Advancing the science of community-level interventions. *American Journal of Public Health* 101: 1410–9. 21680923 10.2105/AJPH.2010.300113 PMC3134512

[R42] Tully LA, Piotrowska PJ, Collins DAJ et al. 2017. Optimising child outcomes from parenting interventions: fathers’ experiences, preferences and barriers to participation. *BMC Public Health* 17: 1–14. 28592244 10.1186/s12889-017-4426-1 PMC5463495

[R43] UNICEF . 2023. Global Health Observatory (GHO) data. Under-five mortality. https://data.unicef.org/topic/child-survival/under-five-mortality/#:∼:text=Globally%2C%20infectious%20diseases%2C%20including%20pneumonia,1990%20to%2038%20in%202021, Accessed 07 November 2023.

[R44] Var C, Oberhelman RA, Shu T et al. 2020. A linked community and health facility intervention to improve newborn health in Cambodia: the NICCI stepped-wedge cluster- randomized controlled trial. *International Journal of Environmental Research & Public Health* 17: 1559. 10.3390/ijerph17051559 PMC7084723 32121288

[R45] Waiswa P, Pariyo G, Kallander K et al. 2015. Effect of the Uganda Newborn Study on care-seeking and care practices: a cluster-randomised controlled trial. *Global Health Action* 8: 24584. 10.3402/gha.v8.24584 PMC4385212 25843498

[R46] Wanjau KN, Muiruri BW, Ayodo E. 2012. Factors affecting provision of service quality in the public health sector: A case of Kenyatta national hospital.

[R47] WorldBank . 2021. World Bank data: Nigeria. https://data.worldbank.org/country/NG, Accessed 10 September 2021.

[R48] World Health Organization . 2008. Quality improvement (QI) in Primary Health Centers - Operations manual for delivery of hiv prevention, care and treatment in high-prevalence, resource-constrained settings. ed. WHO; Kirsty McHarry Sandy Gove, WHO HIV; Mary Lou Lindegren, CDC: Sandra Woods.

[R49] World Health Organization . 2016. Patient engagement: technical series on safer primary care. Geneva: World Health Organization.

[R50] World Health Organization . 2023. The Global Health Observatory: Child mortality and causes of death. https://www.who.int/data/gho/data/themes/topics/topic-details/GHO/child-mortality-and-causes-of-death, Accessed 07 November 2023.

[R51] Woskie LR, Fallah MP. 2019. Overcoming distrust to deliver universal health coverage: lessons from Ebola. *BMJ* 366: l5482. 10.1136/bmj.l5482 PMC6753668 31548212

